# The IFN-λ Genetic Polymorphism Association With the Viral Clearance Induced by Hepatitis C Virus Treatment in Pakistani Patients

**DOI:** 10.5812/hepatmon.15076

**Published:** 2014-03-09

**Authors:** Imran Tipu, Fiona Marriage, Zia-ur-Rahman Farooqi, Hazel Platt, Muhammad Amin Athar, Philip John Day, Andrea Short

**Affiliations:** 1 Institute of Biochemistry and Biotechnology, University of the Punjab, Lahore, Pakistan; 2 Manchester Institute of Biotechnology, University of Manchester, Manchester, UK; 3 Centre for Integrated Genomic Medical Research, University of Manchester, Manchester, UK; 4 National University of Science and Technology, Islamabad, Pakistan

**Keywords:** Polymorphism, Genetic, Polymorphism, Single Nucleotide, Antiviral Agents, Interferons, Ribavirin, Hepacivirus

## Abstract

**Background:**

Polymorphisms in the interferon λ (INF λ) genes on chromosome 19 have been associated with clearance of hepatitis C virus (HCV) induced by interferon and ribavirin therapy however there is no such data available for Pakistani patients with HCV infection.

**Objectives:**

In this study, the effects of single nucleotide polymorphisms (SNPs) have been investigated in response to treatment with interferon-α and ribavirin in a cohort of 75 HCV genotype 3a patients.

**Patients and Methods:**

A total number of 50 SNPs from the Interferon λ region on chromosome 19 were genotyped to investigate allelic associations with the treatment response in HCV type 3a patients. Thirteen SNPs were associated with HCV clearance, with the most significant alleles being RS8109886 (Fisher’s P = 0.0001), RS8113007 (Fisher’s P = 0.0001) and RS12979860 (Fisher’s P = 0.0002).

**Results:**

These SNPs were found to be the most suitable SNPs for predicting treatment response in the present study. These findings support those reported previously. This could be used to improve HCV treatment strategies and suggest that Pakistani patients should be genotyped for the relevant SNPs to identify the patients who are more likely to respond to interferon and ribavirin therapy.

**Conclusions:**

This therapy is costly and can be accompanied by several adverse side-effects, hence pre-treatment prediction of patients who are most likely to benefit would have both economic and patient benefits in the long term.

## 1. Background

Hepatitis C virus (HCV) infection is one of the leading causes of chronic liver disease and has emerged as a global concern of public health, affecting about 3% of the world’s population. Pakistan is the sixth most populated country in the world and has a HCV prevalence rate of 5.9% (1). While there are different subtypes of HCV, genotype 3a is the most common form in patients from Pakistan, with frequency ranging from 28.6% (2) to 89% (3) depending on the province (4). The clinical outcome of HCV infection is determined by the interplay between viral, environmental and host related factors (5). The host’s immune system is the most important factor in viral persistence and innate immunity is the first line of defense, intervening with interferons and natural killer cells (6). This immune response is influenced by genetic polymorphisms in cytokines, their receptors (7) and the polymorphic genetic makeup of human populations. Genetic variations and T-cell responses are responsible for the outcome of HCV treatment (8). The most common type of genetic variations are single nucleotide polymorphisms (SNPs) which occur approximately every 300 nucleotides in the human genome and can be used as biological markers for diseases or conditions. The majority of SNPs have no effect on health, but if SNPs are located within a gene or regulatory region, they can be functional in disease susceptibility and/or treatment response.

Studies have found that infected individuals with same HCV genotype differ in ability to spontaneously resolve infection, even if they have the same ethnic background with similar demographic features and are taking the same IFN-α/ribavirin therapy (9). The host genetics have been identified as key factors in the natural clearance of HCV and host SNPs have been already identified as associated factors in a number of studies in patients from different genetic backgrounds (Table 1). In particular, SNP RS12979860, present 3Kb upstream of the Interleukin 28B gene on chromosome 19, has been associated with a three-fold change in response to treatment against HCV infection in African-American and European cohorts (7). Another SNP from this region of chromosome 19, RS8099917, has been associated with HCV clearance in Australian (10) and Asian populations (11) (Table 1) and is located 8Kb upstream of the IFNL3 gene. In humans, four functional type III IFN λ (IFNL) genes are clustered around this region of chromosome 19encoding cytokines IL29 (IFNL1) , IL28A (IFNL2), IL28B (IFNL3) (12) and IFNL4 (13) and have a number of roles in controlling HCV infection including increasing the antiviral efficacy as a result of increased sub-saturating levels of IFN-α (14). IFN-λ binds to the heterodimeric receptors IFN-λR1 and IL10R2 forming interferon stimulated genes (ISGs) complex and initiating a signal transduction cascade (15) leading to up-regulation of several ISGs with antiviral effects (16). The IFN-λ receptors are present on the plasmacytoid dendritic cells, peripheral B cells, hepatocytes and epithelial cells only so they can be used to target specific cell responses and can also help in avoiding adverse events of INF-α therapy (17). The role of SNPs present in the IFNL3 and IFNL4 genes in the spontaneous clearance of HCV was investigated, in addition to the associative role of SNPs present in the up-and down-stream regions of genes encoding IFN-λ. This data could be of value for predicting the response to interferon and ribavirin therapy in Pakistani patients and though would be of economic and patient benefit in the long term.

**Table 1.** Previous Studies Which Have Reported SNP Allelic Associations

Author	Region	HCV genotype	RS12979860	RS8099917	RS12980275	RS4803219	RS8103142	RS8105790	RS10853728	RS7248668	RS4823221	RS28416813	RS4803217	RS11881222
**Ge et al. (7)**	USA	1	√	√				√						
**Suppiah et al. (10)**	Australia		√											
**Tanaka et al. (11)**	Japan	1		√	√									
**Rauch et al. (18)**	Switzerland	1, 4		√										
**Abe et al. (19)**	Japan			√										
**Mangia et al. (20)**	Italy	2, 3	√											
**McCarthy et al. (21)**	USA		√											
**Thompson et al. (22)**	USA		√											
**Bochud et al. (23)**	Switzerland		√	√	√									
**Smith et al. (24)**	Europe		√	√						√	√			
**Yu.M.Lin et al. (25)**	Taiwan		√											
**Chen et al. (26)**	Taiwan		√	√	√	√		√	√	√				√
**Scherzer et al. (27)**	Austria		√	√										
**Ridruejo et al. (28)**	Argentine	1	√	√										
**Yu et al. (25)**	Taiwan	2				√			√					
**Jun-qiang et al. (29)**	China		√											
**Pedergnana et al. (30)**	Egypt	4	√				√							
**Shi et al. (31)**	China		√	√	√		√							
**de Castellarnau et al. (32)**	Spain		√	√		√	√					√	√	
**Grandi et al. (33)**	Brazil	1	√											
**Prokunina-Olsson et al. (13)**	USA		√											
**Stenkvist et al. (34)**	Sweden		√											
**Gelinas et al. (35)**	France		√	√										
**Ezzikouri et al. (36)**	Morocco		√	√										
**Jung et al. (37)**	Korea		√	√										

## 2. Objectives

In this study, the effects of SNPs have been investigated in response to treatment with interferon-α and ribavirin in a cohort of 75 patients with genotype 3a HCV.

## 3. Patients and Methods

### 3.1. Selection and Description of Participants

Following ethical approval from the Institutional Review Board (University of Punjab, Pakistan) written informed consent for genetic testing including IFN-λ SNPs was obtained from each patient participating in the study. Patients were recruited from different areas of Punjab who visited National Genetics Laboratory, Lahore during March 2010 to May 2011. Patients displaying HCV like symptoms of infection (n = 150) were screened for HCV RNA using an in-house PCR detection technique, of the 150 patients screened, 100 were positive for HCV RNA and 75 were classified as genotype 3a. Each patient was interviewed and a structured questionnaire was completed to figure out the demographic data.

### 3.2. Technical Information

#### 3.2.1. HCV Detection

HCV viral RNA was extracted from the patient’s serum using a QIAamp viral RNA extraction kit (Qiagen). The HCV RNA was detected in 100 individuals using sequence specific primers designed to target the highly conserved 5’ UTR sequence in HCV (Table 2). The viral genotype was detected by nested PCR using unique antisense primers which amplify the 5’ conserved sequence of HCV within the genotype and their poor homology with the sequence derived from other genotypes (Appendix 1). Only 75 patients identified with the HCV genotype 3a were selected for further study, this comprised 75% of the patients screened and thus the study avoided the effect of HCV genotypes on therapy response.

**Table 2.** Significantly Associated SNPs (P < 0.05) With Sustained Virological Response to Interferon and Ribavirin Therapy ^a^

SNPs	MAF	Responder MAF (n = 47)	Non-Responder MAF (n = 28)	OR (95% CI)	P Value
**RS8109886**	0.41	0.32	0.44	3.6 (1.9-6.5)	0.0001
**RS8113007**	0.25	0.19	0.33	3.6 (1.9-6.5)	0.0001
**RS12979860**	0.3	0.23	0.41	3.1 (1.7-5.3)	0.0002
**RS11665818**	0.38	0.29	0.5	2.9 (1.6-5.3)	0.0003
**RS955155**	0.33	0.26	0.38	2.9 (1.6-5.1)	0.0004
**RS688187**	0.31	0.27	0.38	2.7( 1.5-4.7)	0.0011
**RS4803217**	0.3	0.25	0.38	2.7 (1.5-4.7)	0.0011
**RS8105790**	0.19	0.16	0.25	2.6 (1.4-4.6)	0.0022
**RS4803221**	0.22	0.16	0.27	2.6 (1.4-4.6)	0.0022
**RS8099917**	0.19	0.16	0.25	2.6 (1.4-4.6)	0.0022
**RS7248668**	0.19	0.16	0.25	2.6(1.4-4.6)	0.0022
**RS12972991**	0.22	0.17	0.3	2.5(1.4-4.5)	0.0024
**RS11671087**	0.41	0.32	0.5	2.2 (1.2-3.9)	0.0130

^a^ Abbreviations: CI, confidence interval; MAF, minor allele frequency; OR: odds ratio.

#### 3.2.2. Treatment

All patients received three million IU of IFN-α three times a week subcutaneously and ribavirin (10 mg/day/kg body weight) for a total period of six months. Doses of IFN-α were adjusted according to platelet and white blood cell counts of patients. Ribavirin dose varied according to the haemoglobin (Hb) levels and weight of individual patients. The therapy response was monitored by alanine aminotransferase (ALT) and HCV RNA levels at the beginning and end of treatment. The HCV RNA quantification was performed by the Artus HCV RT-PCR (Qiagen) kit using a Rotor-Gene 3000 (Corbett Robotics, Australia) instrument.

#### 3.2.3. DNA Extraction

Human genomic DNA was extracted from peripheral blood mononuclear cells using a QIAamp blood DNA mini kit (Qiagen). DNA was quantified using a Nanodrop-ND1000 spectrophotometer (lab technologies) and concentrations were normalized to 15 ng/µL.

#### 3.2.4. SNP Selection and Genotyping

In total, 50 SNPs were genotyped. Twenty five were from the coding region of the IL28B gene, 23 SNPs covered the 3’ and 5’ UTR’s of all four IFN-λ genes and the remaining two SNPs were from the newly discovered IFNL-4 gene. The details of SNPs are given in supplementary data (Appendix 2 and 3). Genotyping was performed using the iPLEX assay on a SEQUENOM MassARRAY® platform. The primers were designed using the assay designing suite v1.0.1 (SEQUENOM) (Appendix 4). An initial PCR amplified a 50-60 bp region flanking the polymorphic site. The product was treated with 1 U/µL of shrimp alkaline phosphatase at 37˚C for 40 minutes to dephosphorylate any unincorporated dNTPs. The iPLEX reaction product was desalted using a cationic resin, pre-treated with acidic reagents, for optimizing mass spectrophotometric analysis. The desalted iPLEX product was spotted on the SpectroCHIP using a Nano spotter (Sequenom) and loaded on to the mass spectrometer. Each spot was then subjected to a laser under vacuum by the matrix-assisted laser desorption ionization-time-of-flight (MALDI-TOF) method. Assays were designed to SNPs on chromosome 19q13.13 covering the region encoding the IFN-λ genes. After genotyping, SNPs and samples were quality checked.

#### 3.2.5. SNP Quality Controlled

SNPs were excluded from the analyses if the call rate < 90%, Minor Allele Frequencies < 0.05 and the cohort (responders + non-responders) was not in Hardy-Weinberg equilibrium (HWE, P < 0.05). Samples were excluded if the call rate was less than 90%. Call rate, Hardy-Weinberg equilibrium, minor allele frequencies, allelic and haplotypic associations and linkage disequilibrium (LD) were performed using BC|GENE version 3.5-087 software (Biocomputing Platforms, Sweden) whilst Microsoft Excel was used for the determination of means and averages.

### 3.3. Statistics

#### 3.3.1. Association Analyses

Association of the genetic variants and spontaneous HCV clearance, was determined using logistic regression. The major alleles (as RS12979860 C) were compared with minor alleles (rs12979860 T) in statistical analyses to determine odds ratios (OR) and 95% confidence intervals (CI 95%).

#### 3.3.2. Linkage Disequilibrium and Haplotypic Analysis

Linkage disequilibrium between marker loci was assessed and haplotypic blocks were constructed using BC|GENE version 3.5-087 software (Biocomputing platforms, Sweden) and Haploview 4.2 (http://www.broadinstitute.org/haploview/haploview).

#### 3.3.3. Treatment Response

The effectiveness of IFN-λ loci SNPs was estimated for predicting the treatment response by comparing the sensitivity, specificity, positive predictive value (PPV) and negative predictive value (NPV) for minor allele homozygotes. The most clinically useful parameter to investigate the treatment failure is PPV.

## 4. Results

### 4.1. Demographics

Out of 75 patients with genotype 3a HCV enrolled into the study, 46 were male and 29 were female. The virological response was monitored by quantification of HCV RNA at the beginning and at the end of the six months period of the therapy revealing that 63% of subjects (47) showed Sustained Virological Response (SVR) and 37% (28) patients were HCV RNA positive at the end of therapy. It also emerged that 75% of the patients were infected with HCV genotype 3a. These results were consistent with a recent review (4) showing the predominance of genotype 3a in the Pakistani population. The base line demographic, virological and clinical features of patients are shown in Table 3. 

**Table 3.** Demographic and Clinical Characteristics of the Responders and Non-responders to Interferon and Ribavirin Therapy Against HCV Infection ^a^

	Responders	Range	Non Responder	Range
**Number of Patients, No. (%)**	47 (63)		28 (37)	
**Average Age, y**	43	(21-60)	48	(28-63)
**Gender**				
Male	30		16	
Female	17		12	
**Laboratory parameters**				
Hb, g/dL	12.7	(8.2-16.4)	12.8	(7.1-17.1)
WBC, 10×9/L	5.64	(2.8-11)	5.84	(3.3-9.4)
PLT, 10×9/L	232	(93-402)	165	(67-287)
ALT, IU/L	63	(15-224)	93	(38-235)
HCV-RNA, KIU/mL, Initial	1200	(125-9900)	1034	(146-5000)
HCV-RNA, KIU/mL, End of treatment	below threshold	below threshold	2647	(120-9800)

^a^ Abbreviations: ALT, alanine transaminase; Hb, Haemoglobin; PLT: platelets; WBC: white blood cells.

### 4.2. Sample and SNP Quality Control

We analyzed the region of ~ 62.4 kb (Chr 19, nucleotide positions, 39719200-39781600; build GRCh37.p10) containing 50 SNPs (Tables 2 and 4) present in the IFN-λ loci. Out of 50 SNPs, one failed the quality control (QC) criteria and was excluded from the analyses (SNP RS11881222 call rate = 80%); all other samples satisfied the inclusion criteria (> 90% call rate, HWE > 0.05). Twenty four SNPs present in the coding region of the IL28B gene were monomorphic in the studied Pakistani population and were therefore excluded from allelic association and haplotype analysis.

**Table 4.** Haplotypes With Odds Ratios ^a^, ^b^

Haplotype	Frequency, %	Responders, %	Non-responders, %	OR (95% CI)	P Value
**AAATTGCCCATCATG**	58.3	66.0	44.7	2.37 (1.34-4.20)	0.0028
**TCGCCAATGATATGA**	14.0	12.8	17.9	0.68 (0.31-1.48)	0.3286
**AAATTGCCCATAATA**	9.60	7.40	14.0	0.46 (0.18-1.2)	0.106
**TCGCTAATCGCATTG**	8.00	4.20	12.5	0.28 (0.09-0.89)	0.022
**TAGCCAATGGTATGA**	5.30	3.20	7.10	0.41 (0.10-1.64)	0.194
**AAATTGCTCATAATA**	2.00	3.20	1.90	1.52 (0.25-9.27)	0.650

^a^ The odds ratio has been calculated as carrying of haplotype vs. not carrying the haplotype.

^b^ The frequency of six haplotypes in responders and non-responders for a haplotype block covering 13 Kb IFN-λ. The SNP order is RS35790907, RS12972991, RS12980275, RS12982533, RS8105790, RS688187, RS4803217, RS12979860, RS4803221, RS1549928, RS10853727, RS8109886, RS8113007, RS8099917 and RS7248668.

### 4.3. Allelic Association

The allelic association revealed that a region of ~ 39 Kb (Chr 19, nucleotide positions, 39729450-39768250; build GRCh37.p10) containing 13 polymorphic SNPs in Pakistani population is strongly associated (Fisher’s P value = 0.0003-0.0130) with spontaneous clearance and for all of these SNPs, spontaneous HCV clearance was more common with the major alleles. The most significant results were obtained with RS8109886 (Odds ratio of presenting HCV clearance [OR] for C vs. A = 3.6 [95% CI: 1.9-6.5] Fisher’s P = 0.0001), RS8113007 (A vs. T OR = 3.6 [1.9-6.5]; Fisher’s P = 0.0001) and RS12979860 (C vs. T OR = 3.1 [1.7-5.3]; Fisher’s P = 0.0002). Among individuals, taking RS12979860 as an example, the proportion of HCV clearance was much higher in samples with major allele (80% SVR) as compared to minor T allele (34% SVR). The association analysis of response to treatment by IFN-λ SNPs is described in Table 2. 

### 4.4. Linkage Disequilibrium

**Figure 1.**
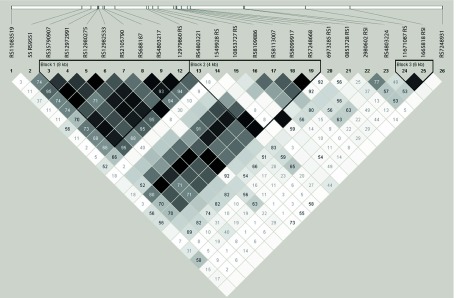
Analysis of Pairwise Linkage Disequilibrium (LD) Plot of IFN-λ Region The linkage disequilibrium between the 17 SNPs in three LD blocks is shown. The red coloured squares represent r^2^ = 1.0 and blue coloured squares represent r^2 ^≤ 0.01.

Estimation of linkage disequilibrium was performed between 23 polymorphic IFN-λ region SNPs, which revealed three haplotypic blocks: haplotype block I, of eight Kb, included eight SNPs (RS35790907, RS12972991, RS12980275, RS12982533, RS8105790, RS688187, RS4803217 and RS12979860) in strong linkage disequilibrium (r^2^ ≥ 0.85) haplotype block II, of 4Kb included seven SNPs (RS4803221, RS1549928, RS10853727, RS109886, RS8113007, RS8099917, RS7248668) in strong linkage disequilibrium (r^2^ ≥ 0.95) and block III contained just two SNPs (RS1671087 and RS11665818) lying approximately 6 kb apart from each other and in strong linkage disequilibrium (r^2^ ≥ 0.85%)(Figure 1). 

### 4.5. Haplotype Analysis

A total number of 6 haplotypes were investigated comprising of 15 SNPs using the Haploview (MIT/Harvard/Brod Institute), among which haplotype one (AAATTGCCCATCATG) comprising of major alleles of 14 SNPs (RS35790907, RS12972991, RS12980275, RS12982533, RS8105790, RS688187, RS4803217, RS12979860, RS4803221, RS1549928, RS10853727, RS8109886, RS8113007, RS8099917 and RS7248668) had most significant association (OR = 2.37, 95% CI = 1.34-4.20, P = 2.8x10-3) with therapy response in comparison with other detected haplotypes. The minor allele frequencies of each haplotype in responders and non-responders to the therapy with their odd ratios are shown in Table 1. 

### 4.6. Treatment Response

The three highly associated SNPs with the treatment response; RS8109886 (PPV of 89%, 95 % CI = 81.17-94.37), RS8113007 (PPV of 74%, 95 % CI = 64.27-82.26) and RS12979860 (PPV of 74%, 95 % CI = 64.27-82.26) are also best indicators for predicting the treatment response. The sensitivity, specificity, prevalence, NPV and PPV of the IFN-λ loci SNPs has been shown in Supplementary Appendix 5.

## 5. Discussion

The treatment of patients with HCV is based on clinical, demographic and virological characteristics of the disease, which are helpful from a population perspective but these baseline parameters are not suitable for predicting the treatment response in HCV patients infected with the most common genotype, 3a. Two SNPs have been most frequently associated with viral clearance across all HCV genotypes in different populations of the world: RS8099917 and RS12979860 (Table 1) and efforts have been largely directed at determining which of them is most likely to be more suitable for establishing the most useful diagnostic test for predicting treatment. Genotype 3a is the most common genotype of HCV infections in Pakistan (4, 38). In a new cohort of 75 type 3a Pakistani patients SNPs in the up-and down-stream regions of IFN-λ and SNPs from IFNL3 and IFNL4 with known association to HCV clearance in other patient populations, were genotyped (Table 1). The allelic associations of four SNPs that have been reported previously in a number of populations were confirmed here (RS8105790, RS12979860, RS8099917 and RS7248668, Table 1) and a novel associations in Pakistani patients was identified (Table 2). The most significant SNPs (RS8109886 and RS8113007) detected by the present this in addition to six other SNPs have not been reported previously to have any association with HCV clearance in other populations and could be relevant to patients of Pakistani origin, although this requires follow-up studies to be fully confirmed.

Five SNPs reported in the literature were excluded from this study (RS4803219, RS8103142, RS4823221, RS28416813 and RS11881222). SNP RS11881222 failed our QC and was excluded for a low call rate (< 80%) but the other five SNPs were not included because the Sequenom primer design software was unable to design suitable primers and probes for them. Excluding these SNPs from our study could represent missed associations in Pakistani patients and constitute additional analyses in this cohort and in future studies to determine whether they have any role in HCV clearance in Pakistani patients as well as the ones reported in patients from Taiwan, Spain, China and Europe (Table 1). None of the SNPs associated with HCV clearance in this study were in coding regions; but were located in regions up-or down-stream of genes or in the 3’ or 5’UTR. This suggests that they have a regulatory function rather than directly affecting protein structure. The 13 SNPs associated with HCV clearance in this study formed 6 haplotypes, of which the major alleles of SNPs RS8109886, RS8113007, RS12979860 and RS8099917 were all present on haplotype I, the haplotype with the highest Odds Ratio for predicting the treatment response (Table 4). The role of these SNPs has been established as having effects on the binding of different transcription factors and alterations of methylation sites resulting in reduced expression of IL28B, and up-regulation of ISGs in the responder haplotypes in response to IFN-α stimulation therapy (24) while IL28B non-responders have high ISG expression in infected hepatocytes, and that high ISG levels independently predicts poor response to the therapy (39). HCV clearance is a complex process, dependent on the type of HCV infection and the host’s immunity-related genetic factors. Some SNPs associated with HCV clearance in Pakistani patients are the same as those that have been detected to have associations in other cohorts too (Table 1) and suggest a common genetic background across multiple populations for HCV clearance. However, number of alleles identified in this study were unique to the present study which could suggest Pakistani-specific factors for HCV clearance, particularly for type 3a. It is important to consider, however, that this data comprised a small sample size and that repeating this study in a larger cohort could affect the findings and alter the outcome of some markers. For this reason, the data presented here should be interpreted with caution until it can be further verified. These findings, however, do support results widely reported from other populations were host genotype has been a proven factor in HCV clearance and treatment response (Table 1). Restricting this analysis to type 3a patients introduced a selection bias meaning if genotyping were to be introduced as a screening strategy, patients would require screening for HCV type prior to genotyping for treatment response. This selection strategy was chosen because type 3a is the most common form of HCV in Pakistan and so represents the largest treatment group. Confirming the association of these SNPs and HCV clearance, in other HCV types requires further investigation. Tailoring treatments to target potential responders, as opposed to generalized, universal treatment strategies, will be of economic benefit but, more importantly, will have substantial benefits for patients, as they would recover quicker and be less likely to require multiple ‘trial-and-error’ treatments. Data from the present study support the associations of SNPs (Table 2) present in the IFN-λ genes with HCV clearance after interferon and ribavirins combined therapy in Pakistani individuals infected with genotype 3a and provide preliminary evidence to suggest patients should be genotyped for the relevant SNPs in order to predict drug response before starting therapy.

## Appendices

**Appendix 1.** The Details of Single Nucleotide Polymorphisms (SNPs) Present in the up- and Down- Stream Region of IFNL-λ Genes. The Annotation of SNPs According to Their Position is Listed With Their Hardy-Weinberg Equilibrium P Values (HW p)

SNP RS No.	SNP Position	Role of SNP	Alleles	HW p
**RS11083519**	chr19:39719263	IFNL3 Downstream	A:T	0.820
**RS955155**	chr19:39729479	IFNL3 Downstream	C:T	0.304
**RS35790907**	chr19:39730755	IFNL3 Downstream	A:T	0.551
**RS12972991**	chr19:39731747	IFNL3 Downstream	A:C	0.831
**RS12980275**	chr19:39731783	IFNL3 Downstream	A:G	0.551
**RS12982533**	chr19:39731904	IFNL3 Downstream	T:C	0.551
**RS8105790**	chr19:39732501	IFNL3 Downstream	T:C	0.906
**RS688187**	chr19:39732752	IFNL3 Downstream	G:A	0.394
**RS4803217**	chr19:39734220	IFNL4 Exon	C:A	0.919
**RS12979860**	chr19:39738787	IFNL4 Intron	C:T	0.173
**RS4803221**	chr19:39739129	IFNL3 Promoter	C:G	1.000
**RS1549928**	chr19:39739709	IFNL3 Promoter	A:G	0.625
**RS10853727**	chr19:39740463	IFNL3 Promoter	T:C	0.118
**RS8109886**	chr19:39742762	IFNL3 Promoter	C:A	0.339
**RS8113007**	chr19:39743103	IFNL3 Promoter	A:T	0.225
**RS8099917**	chr19:39743165	IFNL3 Promoter	T:G	0.906
**RS7248668**	chr19:39743821	IFNL3 Promoter	G:A	0.906
**RS16973285**	chr19:39744696	IFNL3 Promoter	C:T	0.081
**RS10853728**	chr19:39745146	IFNL3 Promoter	G:C	0.387
**RS12980602**	chr19:39752820	IFNL2 Promoter	T:C	0.041
**RS4803224**	chr19:39753014	IFNL2 Promoter	G:C	0.976
**RS11671087**	chr19:39761790	IFNL2 Downstream	T:C	0.122
**RS11665818**	chr19:39768216	IFNL2 Downstream	G:A	0.039
**RS7248931**	chr19:39781583	IFNL1 Promoter	A:G	0.812

**Appendix 2:** . The Details of SNPs Located in IL28B Gene (IFNL-3) Listed According to Amino Acid Position. The Amino Acid Present in Normal (amino acid: context) and Change of Amino Acid Due to Allele Change (amino acid: SNP) Are Listed Accordingly

SNP rs No.	Amino Acid Position.	SNP Position	Amino Acid: Context	Amino Acid: SNP	Allele Change
**RS200289435**	1	chr19:39735606	Methionine	Threonine	ATG→ACG
**RS143935261**	1	chr19:39735607	Methionine	Valine	ATG→GTG
**RS202126177**	2	chr19:39735603	Threonine	Serine	ACC→ATC
**RS630388**	2	chr19:39735602	Threonine	Threonine	ACC→ACT
**RS150569967**	3	chr19:39735601	Glycine	Arginine	GGG→AGG
**RS199952257**	57	chr19:39735438	Lysine	Arginine	AAA→AGA
**RS202143862**	72	chr19:39735101	Arginine	Cysteine	CGC→TGC
**RS145428712**	101	chr19:39734754	Threonine	Methionine	ACG→ATG
**RS200889156**	104	chr19:39734744	Valine	Valine	GTT→GTC
**RS148543092**	108	chr19:39734734	Threonine	Alanine	ACC→GCC
**RS202101632**	108	chr19:39734732	Threonine	Threonine	ACC→ACT
**RS201376760**	114	chr19:39734716	Alanine	Threonine	GCC→ACC
**RS199801376**	116	chr19:39734708	Glycine	Glycine	GGG→GGA
**RS200058568**	123	chr19:39734687	Leucine	Leucine	CTT→CTC
**RS201605224**	126	chr19:39734678	Leucine	Leucine	CTG→CTT
**RS199655870**	132	chr19:39734662	Glutamine	Stop Codon	CAG→TAG
**RS149832972**	133	chr19:39734659	Leucine	Phenylalanine	CTC→TTC
**RS139176035**	134	chr19:39734656	Arginine	Tryptophan	CGG→TGG
**RS201566097**	138	chr19:39734544	Glutamine	Stop Codon	CAG→TAG
**RS145946971**	164	chr19:39734465	Lysine	Threonine	AAG→ACG
**RS143748522**	179	chr19:39734328	Phenylalanine	Valine	TTC→GTC
**RS150748693**	180	chr19:39734325	Arginine	Cysteine	CGC→TGC
**RS201746548**	183	chr19:39734314	Threonine	Threonine	ACG→ACA
**RS200180353**	191	chr19:39734290	Serine	Serine	AGC→AGT
**RS201888594**	194	chr19:39734282	Leucine	Proline	CTG→CCG

**Appendix 3.** The Primers Used for Detection and Genotyping of HCV. (HCF1: HCV Outer Forward Primer, HCR1: HCV Outer Reverse Primer, HCF2: HCV Internal Forward Primer, HCR2: HCV Internal Reverse Primer, HCGF1: HCV Genotype Outer Forward Primer, HCGR1: HCV Outer Reverse Primer, HCGF2: HCV Internal Forward Primer, Rest All Are Specific for Every HCV Genotype With Their Amplified Product Size Using Same Internal Primer

Primer Name	5’-3’ Sequence	Product Size (bp)
**HCF1**	CCCTGTGAGGAACTACTGTCTTCACGC	270
**HCR1**	ACTCGCAAGCACCCTATCAGGCAGTAC	
**HCF2**	AAAGCGTCTAGCCATGGCG	210
**HCR2**	CACAAGGCCTTTCGCGACC	
**HCGF1**	TTGTGGTACTGCCTGATAGGG	470
**HCGR1**	GGATGTACCCCATGAGGATCG	
**HCGF2**	GTGCCCCGGGAGGTCTCGTAG	
**G1a**	ACTCCACCAACGATCTGACC	129
**G1b**	AGCCTTGGGGATAGGTTGTC	233
**G1c**	CTTACCCAAATTGCGTGACC	391
**G2a**	CTCCGAAGTCTTCCTTGTCG	190
**G2b**	AGCAAGTAAACTCCGCCAAC	178
**G2c**	ACCGTTCGGAAGTTTTCCTC	202
**G3a**	ACTCCACCAACGATCTGTCC	258
**G3b**	AGCCTTGGGGATAAGGTGAC	232
**G3c**	GTGACCGCTCGGAAGTCTTA	197
**G4a**	CCGTAAAGAGGCCATGGATA	288
**G5a**	AATCCGCACGTTAGGGTATG	417
**G6a**	CAGCCTTCGCTTCCATAAAG	300

**Appendix 4.** The Primers and Probes Used During the iPLEX Assay on SEQUENOM are Given in Detail With Each SNP Corresponding to the Sequence of Forward and Reverse Primer With the Mass (Daltons) of PCR Product After First PCR. The Extended Product and Mass Represents the Change of Mass With the Different Incorporation of Base and Thus Explaining the Principal Behind the iPLEX Assay. (PCR Mass: Mass of Initial PCR Product. Ext.1 and 2 Products: The Extended Base Which is Complementary to one Present in Initial PCR Product. Ext.1 and 2 Mass: The Masses of Final Products)

SNP ID	Forward Primer	Reverse Primer	PCR Mass	Probe	Ext. 1 Product	Ext.1 Mass	Ext.2 Product	Ext.2 Mass
**RS12979860**	ACGTTGGATGTCGTGCCTGTCGTGTACTGA	ACGTTGGATGAGCGCGGAGTGCAATTCAAC	4563	AGCTCCCCGAAGGCG	C	4810.2	T	4890.1
**RS143748522**	ACGTTGGATGTCCTCCCTACAGGAGTCCC	ACGTTGGATGCAACACAATTCAGGTCTCGC	4752.1	TGTCACCTTCAACCTC	C	5039.3	A	5079.2
**RS201566097**	ACGTTGGATGTGAGCAGCGTCCTTCCCCTG	ACGTTGGATGGTCCTGGGCCCTGCCGTG	5115.3	GACTCTGCCCACAGATC	G	5362.5	A	5442.4
**RS148543092**	ACGTTGGATGTGGTCCAAGACATCCCCCAG	ACGTTGGATGCCTGACGCTGAAGGTTCTG	5242.4	CTGGTCAGTGTCAGCGG	C	5489.6	T	5569.5
**RS11881222**	ACGTTGGATGCACACCTGCTACCCCTTCC	ACGTTGGATGGGAACAAGTGAAGGTGACAG	5282.4	ACCCCTTCCCTCTGCTCC	G	5529.6	A	5609.5
**RS8105790**	ACGTTGGATGCTTCCTGACATCACTCCAAT	ACGTTGGATGGTCAGCATCATTAGCGGAAG	5394.5	CATCACTCCAATGTCCTG	C	5641.7	T	5721.6
**RS202126177**	ACGTTGGATGGCTCCCTTTCTCTCTGTGAC	ACGTTGGATGACAGGAACTGCTCCAGTCAC	5796.8	CTCTGTGACACAGACATGA	G	6044	C	6084
**RS150748693**	ACGTTGGATGAGGCCTCTGTCACCTTCAAC	ACGTTGGATGTTGCATGACTGGCGGAAGG	5938.9	CTGTCACCTTCAACCTCTTC	G	6186.1	A	6266
**RS11665818**	ACGTTGGATGAAGAAAGACCTCCACCATGC	ACGTTGGATGAGTCACCCCTATTTCCTAGC	5947.9	TTATCATCTGCCCCCAACTC	A	6219.1	G	6235.1
**RS4803221**	ACGTTGGATGTCCTGTGCACGGTGATCGC	ACGTTGGATGTCCCTCAGCGCCTTGGCAG	6319.1	CCCAAGGCGCTGCCTGCTCTC	G	6566.3	C	6606.3
**RS199801376**	ACGTTGGATGATATGGTGCAGGGTGTGAAG	ACGTTGGATGCCTGACGCTGAAGGTTCTG	6456.2	ACGGGGCTGGTCCAAGACATC	C	6703.4	T	6783.3
**RS12980602**	ACGTTGGATGTACTTTATTAAGTGGTAAAC	ACGTTGGATGCTCTGGTTTTTGTTCATCTG	6505.3	GAACAATATGAAAGCCAGAGA	C	6752.5	T	6832.4
**RS1549928**	ACGTTGGATGTGCCCTCCAACACTCGGTTT	ACGTTGGATGCGAAGATAAAGACAACCAGG	6643.3	GCCTAATTGTCTCTGTCCCTGT	G	6890.5	A	6970.4
**RS688187**	ACGTTGGATGTCTAGCACGAATCCATTAC	ACGTTGGATGCTTTTGGTAACAGTCACAAG	6651.4	GCACATGCAGCAACACACCACA	A	6922.6	G	6938.6
**RS12980275**	ACGTTGGATGTTCCTATTAACCCCTCCCGC	ACGTTGGATGATGAGGTGCTGAGAGAAGTC	7016.6	ACCGGCAAATATTTAGACACGTC	G	7263.8	A	7343.7
**RS11671087**	ACGTTGGATGAAGCTCCTTTGCCGAGTAAC	ACGTTGGATGGAAGATGCCACCCCAAAGTC	7056.6	CCTGTGCCGAGTAACATAAGATA	C	7303.8	T	7383.7
**RS139176035**	ACGTTGGATGTTCACACCCTGCACCATATC	ACGTTGGATGTGCTCAGAGCTCACAGACCT	7168.7	CTGAACCATATCCTCTCCCAGCTC	G	7415.9	A	7495.8
**RS4803217**	ACGTTGGATGATAAATAGCGACTGGGTGAC	ACGTTGGATGCCAGTCATGCAACCTGAGAT	7449.9	GCGACTGGGTGACAATAAATTAAG	C	7697.1	A	7721.1
**RS7248668**	ACGTTGGATGGAGTGGCGATTGTGCCACTA	ACGTTGGATGCTTTTGCAGAGCAGAGGTTG	7552.9	CCCAGATTGTGCCACTACTATGCTC	G	7800.1	A	7880
**RS16973285**	ACGTTGGATGTGCACGTTTCATTTGTTTA	ACGTTGGATGCCCCACCCATCTTAAGCATC	7593.9	CACGTTTCATTTGTTTATTGATTTC	C	7841.1	T	7921
**RS12982533**	ACGTTGGATGAAGAGAGTTCTGGAGATTGC	ACGTTGGATGTTACAGGTCTGGTCCTAGTG	7762	GGGTGAGATTGCTTGCCGAACAATG	C	8009.2	T	8089.1
**RS8113007**	ACGTTGGATGACAAAAGGAGGAACAGTGAC	ACGTTGGATGGGAGAGTTAAAGTAAGTCTTG	7960.2	TGACAAATTGTTAAAAAATATTTACC	T	8231.4	A	8287.3
**RS201605224**	ACGTTGGATGATGTCTTGGACCAGCCCCTT	ACGTTGGATGGGCCCTGACGACTCACACA	8132.3	TGTCGTGGACCAGCCCCTTCACACCCT	C	8419.5	A	8459.4
**RS7248931**	ACGTTGGATGCTCATCATCTCAAGAACTAGG	ACGTTGGATGGTTGGCATCTATTGATTGGC	8333.5	GATATCAAGAACTAGGAAAATCTCAAG	G	8580.7	A	8660.6
**RS200058568**	ACGTTGGATGCTGACACTGACCCAGCCCT	ACGTTGGATGGGCCCTGACGACTCACACA	4488.9	CTTGGACCAGCCCCT	G	4736.1	A	4816
**RS202101632**	ACGTTGGATGCCTGACGCTGAAGGTTCTG	ACGTTGGATGTGGTCCAAGACATCCCCCAG	4609	GGTTCTGGAGGCCAC	G	4856.2	A	4936.1
**RS145946971**	ACGTTGGATGCCGCCTCCACCATTGGCTG	ACGTTGGATGAGACCTCAGTCCCTCTCTTC	4893.2	CAGGAGGCCCCAAAAA	G	5140.4	T	5164.4
**RS202143862**	ACGTTGGATGTCTCACCTGCAGCTGCCTCA	ACGTTGGATGCCTTTGCTGTCTAGGAAGAG	5036.3	TGGAAGAGGCGGGAGC	A	5307.5	G	5323.5
**RS201376760**	ACGTTGGATGCCTGACGCTGAAGGTTCTG	ACGTTGGATGAAGGGGCTGGTCCAAGACAT	5100.3	CCGCTGACACTGACCCA	T	5371.5	C	5387.5
**RS630388**	ACGTTGGATGGCTCCCTTTCTCTCTGTGAC	ACGTTGGATGACAGGAACTGCTCCAGTCAC	5203.4	TGTGACACAGACATGAC	G	5450.6	A	5530.5
**RS955155**	ACGTTGGATGAACTATGGGCCAACACTGTC	ACGTTGGATGACTGGTATGTCAGCTCCTCG	5211.4	TGTGCACTGAGGGCCCA	T	5482.6	C	5498.6
**RS10853728**	ACGTTGGATGAGACAGACTCTCATCCTCAC	ACGTTGGATGTCCATTTCCATTCTGTCTCG	5676.7	CCATCCTCACCAAAGCTTA	G	5923.9	C	5963.9
**RS201746548**	ACGTTGGATGAGGTTGCATGACTGGCGGAA	ACGTTGGATGCCTCTGTCACCTTCAACCTC	5756.8	CAACACAATTCAGGTCTCG	C	6004	T	6083.9
**RS199952257**	ACGTTGGATGATGGTGACCCTTGGAGTGC	ACGTTGGATGAGGAGCTGCAGGCCTTTAAG	5787.8	TGGACTCACTAAGGCATCT	C	6035	T	6114.9
**RS35790907**	ACGTTGGATGACATGTCTGAGAGCCGAATC	ACGTTGGATGTCTTCTGCCAGGTTAGAAGC	5885.9	GCTGTACAGGTGAGAACAA	A	6157.1	T	6212.9
**RS200180353**	ACGTTGGATGTCTCAGGTTGCATGACTGGC	ACGTTGGATGCTCACGCGAGACCTGAATTG	6336.1	CTTGCAGACACACAGGTCCCC	A	6607.3	G	6623.3
**RS8099917**	ACGTTGGATGCAATTTGTCACTGTTCCTCC	ACGTTGGATGACTGTATACAGCATGGTTCC	6368.1	TTTTTCCTTTCTGTGAGCAAT	G	6655.4	T	6695.2
**RS11083519**	ACGTTGGATGCAAAGCCAACTCAATTGAGG	ACGTTGGATGTTGTGATCCACTTTTCTGCC	6460.2	TTGAGGAAGAATAGCCTTTTC	A	6731.4	T	6787.3
**RS10853727**	ACGTTGGATGACGCTCACCATTTGCTGAAC	ACGTTGGATGATGTAAGCATGCGCAGAGAG	6825.5	GAAGACATCATATGAAGAGGCA	C	7072.7	T	7152.6
**RS4803224**	ACGTTGGATGTAGTCCCTAAGCAGCTGGAG	ACGTTGGATGAACAGAGTGAGACCCCCATC	6994.5	GCTTGAGCTGCAGGCACCCACCA	G	7241.7	C	7281.8
**RS200889156**	ACGTTGGATGCCGTGGCTTTGGAGGCTGA	ACGTTGGATGTGGTCCAAGACATCCCCCAG	4593	CCTGACGCTGAAGGT	G	4840.2	A	4920.1
**RS8109886**	ACGTTGGATGTTCCTGTCTCTGTCTCTGGC	ACGTTGGATGTTGATTGAGACAGACAGAGC	4810.2	TCCAACAAGCATCCTG	C	5057.3	A	5081.4
**RS200289435**	ACGTTGGATGGCTCCCTTTCTCTCTGTGAC	ACGTTGGATGACAGGAACTGCTCCAGTCAC	5154.4	TCTCTGTGACACAGACA	G	5401.6	A	5481.5
**RS201888594**	ACGTTGGATGATCTCAGGTTGCATGACTGG	ACGTTGGATGGCGAGACCTGAATTGTGTTG	5253.4	GGAAGGGTCAGACACAC	A	5524.6	G	5540.6
**RS199655870**	ACGTTGGATGATGTCTTGGACCAGCCCCTT	ACGTTGGATGGGCCCTGACGACTCACACA	5355.5	CGGCACCATATCCTCTCC	G	5602.7	T	5626.7
**RS12972991**	ACGTTGGATGGGAATTTGACTTCTCTCAGC	ACGTTGGATGCAGTGAAATAAGCCAGTCTC	5435.5	GGCTCTCAGCACCTCATG	C	5722.7	A	5762.6
**RS149832972**	ACGTTGGATGGGCCCTGACGACTCACACA	ACGTTGGATGATGTCTTGGACCAGCCCCTT	4547	TCACACAGGCCCGGA	A	4818.2	G	4834.2
**RS143935261**	ACGTTGGATGGCTCCCTTTCTCTCTGTGAC	ACGTTGGATGACAGGAACTGCTCCAGTCAC	5130.4	CTCTCTGTGACACAGAC	T	5401.6	C	5417.6
**RS145428712**	ACGTTGGATGAAGGGGCTGGTCCAAGACAT	ACGTTGGATGCCGTGGCTTTGGAGGCTGA	4786.1	CTCCAGAACCTTCAGC	A	5057.3	G	5073.3
**RS150569967**	ACGTTGGATGTTTCTCTCTGTGACACAGAC	ACGTTGGATGACAGGAACTGCTCCAGTCAC	4859.2	TGACACAGACATGACC	T	5130.4	C	5146.4

**Appendix 5.** Prediction of Response to Therapy With Homozygous Responder IFN-λ Region SNPs ^a^

SNP	Sensitivity, % (95% CI)	Specificity, % (95%CI)	Prevalence, % (95%CI)	PPV, % (95%CI)	NPV, % (95%CI)
**RS955155**	51.39 (42.92-59.79)	53.57 (39.75-67.01)	72.00 (65.23-78.10)	74.00 (64.27-82.26)	30.00 (21.24-39.98)
**RS12972991**	59.35 (50.12-68.11)	64.94 (53.21-75.46)	61.50 (54.38-68.28)	73.00 (63.20-81.39)	50.00 (39.83-60.17)
**RS8105790**	57.14 (48.28-65.68)	64.18 (51.53-75.53)	66.50 (59.50-73.00)	76.00 (66.43-83.97)	43.00 (33.14-53.29)
**RS688187**	52.48 (43.91-60.95)	55.93 (42.40-68.84)	70.50 (63.66-76.72)	74.00 (64.27-82.26)	33.00 (23.92-82.26)
**RS4803217**	52.48 (43.91-60.95)	55.93 (42.40-68.84)	70.50 (63.66-76.72)	74.00 (64.27-82.26)	33.00 (23.92-82.26)
**RS12979860**	56.92 (47.95-65.57)	62.86 (50.48-74.11)	65 (57.95-71.59)	74.00 (64.27-82.26)	44.00 (34.08-54.28)
**RS4803221**	51.23 (42.80-60.04)	53.23 (40.12- 66.01)	69.00 (62.09-75.33)	71.00 (61.07-79.64)	33.00 (23.92-43.12)
**RS8109886**	60.09 (52.55-68.92)	79.63 (66.47-89.35)	73.00 (66.28-79.02)	89.00 (81.17-94.37)	43.00 (33.14-53.29)
**RS8113007**	52.48 (43.91-60.95)	55.93 (42.40-68.84)	70.05 (63.66-76.72)	74.00 (64.27-82.26)	33.00 (23.92-43.12)
**RS8099917**	42.01 (34.47-49.83)	6.45 (0.98-21.26)	84.50 (78.73-89.22)	71.00 (61.07-79.64)	2.00 (0.30-7.05)
**RS7248668**	42.01 (34.47-49.83)	6.45 (0.98-21.26)	84.50 (78.73-89.22)	71.00 (61.07-79.64)	2.00 (0.30-7.05)
**RS11671087**	59.35 (50.12-68.11)	64.94 (53.21-75.46)	61.50 (54.38-68.28)	73.00 (63.20-81.39)	50.00 (39.20-81.39)
**RS11665818**	62.30 (53.07-70.91)	69.23 (57.76-79.19)	61.00 (53.87-67.80)	76.00 (66.43-83.97)	54.00 (43.74-64.01)

^a^ Abbreviations: 95% CI, 95% confidence interval; PPV, Positive predictive value; NPV, Negative predictive value
